# Homework adherence in mindfulness-based cognitive interventions for female sexual dysfunction: a scoping review

**DOI:** 10.1093/jsxmed/qdae108

**Published:** 2024-09-12

**Authors:** Fernanda Rafaela Cabral Bonato, Nicolas de Oliveira Cardoso, Lori A Brotto

**Affiliations:** Graduate Program in Psychology, Human Science, Federal University of Paraná (UFPR), Curitiba, Brazil; Graduate Program in Psychology, School of Health and Life Sciences, Pontifical Catholic University of Rio Grande do Sul (PUCRS), Porto Alegre, Brazil; Department of Obstetrics and Gynecology, University of British Columbia, Vancouver, British Columbia, Canada

**Keywords:** homework, mindfulness, mindfulness-based cognitive therapy, female sexual dysfunction

## Abstract

**Background:**

Mindfulness-based cognitive therapy (MBCT) includes regular home practice of mindfulness exercises as a key means of cultivating mindfulness. Although there are instruments available for measuring homework adherence following cognitive behavioral therapy (CBT), little is known about measuring homework practice in MBCT studies for sexual dysfunction.

**Aim:**

In this review we sought to investigate which items and instruments are the most used for the assessment of homework adherence in studies evaluating MBCT for the treatment of female sexual dysfunction (FSD). We also investigated the types of homework used in these interventions.

**Methods:**

Five databases (PubMed, Scopus, PsycINFO, Embase, and Web of Science) were searched and a total of 30 articles were included in this review.

**Outcomes:**

Our main findings revealed that there was no gold standard instrument used to assess homework adherence in patients using MBCT interventions for FSD, and that most of the reported studies did not provide information on how they assess homework adherence.

**Results:**

Six of the 9 studies for which the articles reported how homework was assessed used only ad hoc measures. Only 3 studies used psychometrically validated instruments. We also found that mindfulness, psychoeducation, and CBT exercises were the most prescribed homework.

**Strengths and Limitations:**

This review uniquely integrates homework adherence measures with studies on FSD that evaluate mindfulness, finding no gold standard for assessing adherence. However, limitations including both MBCT and CBT interventions, limiting generalization to MBCT alone, the predominance of Western-based studies, and the lack of reporting on instruments used to assess adherence, indicating a gap in the field.

**Conclusion:**

Further studies should consider adapting existing instruments that assess homework adherence in studies of CBT for other psychopathologies or seek to develop new psychometrically validated instruments for MBCT interventions that assess homework adherence.

## Introduction

Sexual health is considered a physical, emotional, mental, and social aspect of well-being and not merely the absence of disease or dysfunction.^1^ On the other hand, “sexual dysfunctions” are a heterogeneous group of disorders that are typically characterized by clinically significant disturbance in a person’s ability to respond sexually or to experience sexual pleasure. Sexual dysfunctions involve interactions between biological, sociocultural, and psychological factors and are ordinarily divided into male and female disorders.^2^ In general, sexual dysfunctions in women fall into the categories of low or absent desire, difficulties with or inability to orgasm, and genito-pelvic pain.^3^

The psychological mechanisms of female sexual dysfunction (FSD) are complex and multifaceted, involving emotional (eg, anxiety, depression, stress), cognitive (eg, negative beliefs and attitudes about sex, unrealistic expectations, body image issues), and behavioral factors (eg, relational factors, lack of communication with partner, avoidance of intimacy).^4^ Given the established role of these factors in maintaining sexual difficulty, there is a strong need for evidence-based psychological treatments that address these issues as a means of improving FSD.^4^ Psychological treatments remain the gold standard for treating FSD.^5–7^ In recent years, mindfulness-based interventions have shown significant efficacy in treating FSD, including substantial improvements in various aspects of the sexual response cycle that are maintained in the long term. Furthermore, these interventions also reduce symptoms of depression and anxiety and contribute to positive changes in many areas, such as couple harmony and communication.^8^^,^^9^

Mindfulness has its roots in Eastern contemplative traditions like meditation^10^ and involves three dimensions: attention, intention, and attitude. Of note, however, mindfulness is the secular and somewhat simplified adaptation of meditation and has been readily taken up in Western society.^10^ Paying attention consists of observing the operations of internal and external experiences from moment to moment. At the same time, intention concerns observing yourself and developing self-regulation, self-exploration, and self-liberation, bringing attention to yourself, your thoughts, and your body. Mindfulness can also be defined as an attentive, nonjudgmental focus on experiences in the here and now.^10^^,^^11^

Previously, research has shown that sexual beliefs and cognitive distraction during sexual activity are prevalent among women with sexual dysfunction and have negative impacts on their sexual satisfaction and desire.^12^^,^^13^ On the other hand, mindfulness practices help to improve mood and focus, encouraging acceptance of the present moment, decreasing the tendency to self-criticize, and helping with distracting thoughts.^14^^,^^15^

Mindfulness-based cognitive therapy (MBCT) has been tested extensively as a treatment for various FSDs, such as sexual interest/arousal disorder, provoked vestibulodynia, orgasm disorders, and sexual problems in cancer survivors, patients who have a history of childhood sexual abuse, and patients with sexual problems after multiple sclerosis.^16–20^

Delivery of MBCT treatment typically includes homework related to regular daily practice of mindfulness exercises aimed at increasing self-awareness of bodily changes, as well as the thoughts that arise in the present moment, in nonsexual and sexual scenarios.^15^^,^^21^^,^^22^ Homework has been emphasized as an essential component of treatment that is required to develop and hone the skills involved in mindfulness and to see symptom improvement in conditions like depression, anxiety, and FSD.^23^^,^^24^ This is especially true for FSD, because practicing mindfulness at home increases awareness of feelings during sexual intercourse, improves communication, reduces anxiety, and develops new sexual skills.^23^^,^^24^ The therapist plays an active role in recommending homework that will provide opportunities to practice and incorporate newly acquired skills.^25^ In this manner, homework extends the learning experience from the office to the home, augmenting the overall therapeutic benefits.^25^ Typically, homework assignments from sex therapists contain four basic components: psychoeducation, bibliotherapy, communication skills, and physical assignments.^25^ The last two are commonly prescribed to be performed with a sexual partner, aiming to reduce misinformation about sexual function and negative attitudes that have been internalized, including past negative experiences that contribute to FSD.^25^

Despite the critical role that homework plays in cultivating mindfulness skills, few studies have systematically documented what homework was given, how it was measured, and whether the partner was involved in the homework. Furthermore, many homework assignments are recommended by MBCT studies, but it is not clear which homework is most used. In addition, homework is often evaluated with ad hoc instruments that are not psychometrically validated.^13–32^ The use of nonvalidated instruments should be avoided due to the difficulty in comparing results across studies and the potential for conclusions to be made about the efficacy of a program even when the amount of patient engagement is unknown.^33–35^ Additionally, the development of new measurement instruments, even those with strong psychometric evidence, requires a solid theoretical foundation that justifies their necessity.^33–35^ Therefore, a literature review and confirmation of the absence of similar existing instruments are the initial steps recommended by some best practice guidelines for instrument development.^33^^,^^36^

Considering the importance of homework for cultivating the skills of mindfulness, as well as the use of ad hoc instruments by various researchers in this field of study, in this review we aimed to investigate which are the most used items and instruments for the assessment of homework adherence in studies evaluating MBCT for the treatment of FSD. We also investigated the types of homework used in these interventions.

Due to the interchangeable use of the terms homework adherence, compliance, and completion in various studies, in the present study we elected to use only the term adherence, which is an active process whereby patients seek behavioral changes, taking responsibility for their own overall well-being.^37^

The goal of this scoping review was to investigate which are the most used items and instruments for the assessment of homework adherence in studies evaluating MBCT for the treatment of FSD.

## Methods

To perform this scoping review we followed the checklist proposed in the Preferred Reporting Items for Systematic Reviews and Meta-analyses extension for Scoping Reviews^38^ (PRISMA-ScR).

### Search strategy

The search for papers was conducted by two independent researchers (N.D.O.C. and F.R.C.B.) in January 2024. The search string was developed by N.D.O.C. and F.R.C.B. according to the search terms and concepts recommended by two vocabulary databases: Thesaurus (PsycINFO) and Medical Subject Headings (MeSH/PubMed). We also consulted the keywords used by some previous highly cited studies in the area of female sexuality.^39^^,^^40^ The final string was also approved by the senior author (L.A.B.).

The following keyword string was used in all searches: (“female sexual dysfunction” or “female sexual disorder” or “sexual function” or “sexual function disturbance*” or ``dyspareunia'' or “hypoactive sexual desire” or “orgasmic disorder” or “orgasmic dysfunction” or “psychological sexual dysfunction” or ``vulvodynia'' or ``vestibulodynia'' or “vulva* pain” or ``vaginismus'' or “genital pelvic pain” or “sexual interest/arousal disorder” or “vulvar disease” or ``anorgasmia'' or “sexual aversion disorder” or “sexual arousal disorder” or “sexual pain disorder” or “sexual pain/penetration disorder” or “sexual pain” or “female pelvic floor dysfunction” or “female pelvic pain”) and (``mindfulness''). The searches were performed in five databases: PsycINFO, Scopus, Web of Science, Embase, and PubMed. The only filter used in all databases was document type (ie, article or journal article).

### Data selection and eligibility

The articles located in the databases were imported to the Rayyan website, where the selection and screening steps were conducted. Rayyan is a free tool that helps researchers perform systematic or scoping reviews. This tool has a “blind mode” (ie, similar to the peer-review process) that reduces the risk of selection bias.^41^ Then, after completion of the title and abstract–screening process, the website allows for the removal of blind mode, showing the decisions made by each researcher. Then, a new tab named “conflicts” appears, which shows all articles with different decisions regarding inclusion and exclusion of articles in this review.

In the present study, after duplicate papers were excluded, the remaining studies were screened by two independent judges (N.D.O.C and F.R.C.B.). The senior author (L.A.B.) resolved any disagreements between raters.

The inclusion criteria used in the screening of published study reports were the following: (1) The reported study included women aged ≥18 years with an established diagnosis of any FSD or report of any “sexual issue,” which was defined as anything women reported feeling distressed about regarding their sexuality that did not meet the full diagnostic criteria for FSD.^42^ (2) The study included least one group of women who received MBCT and homework was mentioned in the publication. (3) A measure was reported that was used in the study to assess homework adherence and the homework prescribed was also reported. (4) The study was a randomized clinical trial (RCT) or a nonrandomized clinical trial (NRCT) consisting of one group (pre-/post-test) or multiple groups (eg, intervention and control), regardless of the level of blinding. Exclusion criteria were the following: (1) duplicate papers; (2) studies with heterogeneous samples in the same intervention group (eg, men and women/couples); (3) studies that primarily delivered another intervention with a smaller degree of mindfulness (eg, CBT intervention studies); (4) publications with secondary analysis of previous clinical trials. There were no restrictions on the year or language of the publications.

### Data extraction

The following data were extracted from the included papers by two independent reviewers (N.D.O.C and F.R.C.B.): (1) identification of the study (last name of the author, country, and publication year); (2) study design (RCT or NRCT); (3) number and length of intervention sessions; (4) homework assessment instruments; (5) partner involvement during homework; (6) homework prescribed; (7) homework duration (number and length of home practices); (8) sample characteristics (eg, sample size, mean age, sexual dysfunction). All data extracted were reviewed by the senior author.

### Data synthesis

The results section was divided into three categories: (1) publication and sample related information, (2) homework prescribed, and (3) instruments used to assess homework adherence. Those categories were developed inductively according to data extraction and refined through critical analysis. Furthermore, some studies used different nomenclatures to refer to the same technique; these nomenclatures were unified to avoid overlap during the presentation and discussion of the results. For example, the home practices of “mindfulness” and “meditation” were grouped into the category “mindfulness”.

## Results

**Figure 1:**
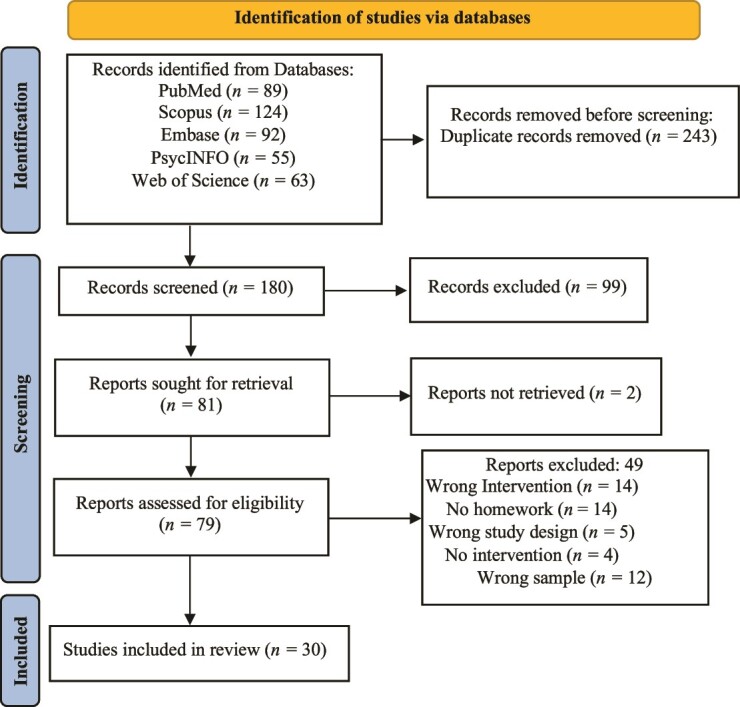
Paper selection flowchart prepared using the Preferred Reporting Items for Systematic Reviews and Meta-analyses extension for Scoping Reviews (PRISMA-ScR).^38^

Initially we identified 423 papers in the 5 assessed databases. After duplicates were removed, the total number of records screened was 180. Next, 99 papers were excluded after title and abstract screening. The main reasons for exclusion at that point were the following: being off topic (not addressing FSD or not assessing homework) and (2) incompatible method (eg, literature reviews, clinical trial registration protocols). After the screening stage, 81 studies that met our inclusion criteria were selected for full-text review. The papers most commonly excluded at the full-text review stage were papers that did not provide any information regarding the use of homework, did not evaluate MBCT, were not NRCTs or RCTs, reported delivery of couples therapy that integrated MBCT, or evaluated a standard CBT intervention. During the full-text review, there was disagreement among the raters regarding the relevance of 11 of the 80 studies (IRR = 0.86); those inconsistencies were resolved through discussion. All of the research team members were in agreement regarding the final inclusion of 30 papers. Figure 1 shows the steps of data selection according to the PRISMA-ScR.^38^

Table 1 presents the main characteristics of the included studies. Each article has been assigned a number (eg, 1, 2, 3...). These numbers are used to refer to the included studies throughout the results section. Table 2 lists the main characteristics of the participants.

**Table 1:** Characteristics of included studies and homework prescribed.

**Reference and country**	**Study design**	**Homework prescribed**	**Homework with partner (yes/no)**	**Homework assessment**	**Homework duration, No. of sessions** ^a^	**Homework session duration, min** ^b^
1. Brotto et al. (2008a): Canada	NRCT: 3 sessions spaced 2 wk apart, 90 min each	1) Psychoeducation: reading, 2) Mindfulness: everyday life, self-observation, body awareness, and 3) CBT: monitoring sexual beliefs, communication, behavior exercises and cognitive challenging	Yes	NR	NR	NR
2. Brotto et al. (2008b): Canada	NRCT: 3 sessions, 60 min each	1) Psychoeducation: body image and intimate relationship	Yes	NR	NR	NR
3. Brotto et al. (2012): Canada	RCT: 2 individual assessments (pre- and posttreatment), 60 min each, and 2 treatment sessions delivered in a group format, 120 min at the first session and 60 min at the second session	1) Psychoeducation: evaluation of factors contributing to sexual experiences, sexual response cycle, 2) Mindfulness: observation and body awareness; and 3) CBT: skills in nonsexual and sexual situations	NR	NR	NR	NR
4. Brotto & Basson (2014): Canada	NRCT: 4 sessions, 90 min each	1) Psychoeducation; body image worksheets, completing a worksheet that considered the possible contributors to their sexual desire/arousal complaints, 2) Mindfulness: daily practice, mindfulness of breath, body scan with and without genitals, sensate focus, and 3) CBT: monitoring sexual beliefs	Yes	Ad hoc Likert scale from 0 (did not complete homework/did not attend or participate in session) to 2 (notable efforts at completing homework/attending session)	NR	NR
5. Bober et al. (2015): USA	NRCT: 1 session, 210 min	1) Psychoeducation: information about vaginal dilators, about sexual health, websites, and books, 2) Mindfulness: body scan, and 3) CBT: muscle relaxation	NR	NR	NR	NR
6. Brotto et al. (2015): Canada	NRCT: 4 sessions, NR session duration	1) Psychoeducation: think about the timeline and sequelae of their PVD, sexual response cycle, 2) Mindfulness: daily practice, body scan, and 3) CBT: completing one though record	NR	NR	NR	NR
7. Brotto et al. (2016): Canada	NRCT: 4 sessions, NR session duration	1) Mindfulness: daily practice, body scan, seeing meditation, mindfulness of genitals, sensate focus, 2) CBT: documenting thoughts, emotions, or beliefs	NR	Ad hoc - Likert scale from 0 (did not complete homework/did not attend sessions) to 2 (notable efforts at completing homework/attending sessions)	NR	NR
8. Kanter et al. (2016): USA	RCT: 7 session, 120 min each	1) Mindfulness: daily practice	NR	NR	NR	NR
9. Paterson et al. (2017): Canada	NRCT: 8 sessions, 135 min each	1) Psychoeducation: daily homework included reading and thinking about the causes of their sexual dysfunction, sections on communication practices with a partner, and 2) Mindfulness: mindful eating, body scan, stretch and breath, sitting meditation, working with difficulty and meditation of their choosing	NR	Ad hoc - Likert scale from 0 (did not complete homework) to 2 (notable efforts at completing homework)	±49	± 27.5 daily
10. Gunst et al. (2018): Finland	RCT: 4 sessions, 75 or 90 min each	1) Psychoeducation: body image worksheets, completing a worksheet that considered the possible contributors to their sexual desire/arousal complaints, 2) Mindfulness: daily practice, mindfulness of breath, body scan with and without genitals, sensate focus, and 3) CBT: monitoring sexual beliefs	NR	Ad hoc - Likert scale from 1 (Did not follow homework instructions at all) to 5 (Followed homework instructions exactly as instructed)	NR	NR
11. Mosalanejad et al. (2018): Iran	RCT: 3 groups: PLMEG: 16 sessions, approximately 10 min per day; - M: 8 sessions, 90 min per day, and - PLMEG + M: 8 sessions, NR session duration.	1) Mindfulness: body scan, mindful in different context such eating, routine activities, breathing, sitting meditation, be awareness of consumption	NR	NR	NR	NR
12. Brotto et al. (2019): Canada	RCT: 8 sessions, 135 min each	1) Mindfulness: daily practice exercises with audio-recorded mindfulness practices to follow at home	NR	A modified version of the MBCT Adherence Scale + Ad hoc - Likert scale from 0 (not at all) to 4 (high degree) in two questions: (i) overall, to what degree were you able to complete your homework assignments? and (ii) overall, since the last group session, to what degree have you been able to practice the skills and exercises you learned in group.	NR	20-45 daily
13. Guillet, et al. (2019): USA	RCT: Two groups: -M-gCBT: 8 sessions, 150 min each, and - ES: 3 sessions, 90 min each, throughout the 8 wk	1) Mindfulness: body scan, mindfulness of breath and radical acceptance meditations	NR	NR	NR	NR
					NR	ES: 60 weekly
14. Van Driel et al. (2019): Netherlands	RCT: MBSR: 8 sessions, 150 min each	1) Mindfulness: exercises provided on a MP3 Player	NR	NR	±42	30-45 daily
15. Adam et al. (2020): Belgium	RCT: Self-administered treatment, compost by 7 videos, NR duration	1) Psychoeducation: and watching videos about orgasm, thinking about erotic context and erotic foreplay, 2) Mindfulness: practiced 3- to 12-minute-long mindfulness meditation exercises, sensate focus, and 3) CBT: documenting thoughts, emotions and beliefs	Yes	NR	NR	NR
16. Bober et al. (2020): USA	NRCT: 1 session, 240 min	1) Psychoeducation: take-home materials, including instructions for exercises, and information about personal products and relevant resources	NR	NR	NR	NR
17. Brotto et al. (2021): Canada	RCT: 8 sessions, 135 min each	1) Mindfulness: body scan, stretch and breath, sitting meditation, working with difficulty, walking meditation and meditation of their choosing	NR	MBCT Adherence Scale	NR	138 weekly
18. Gorman et al. (2021): USA	NRCT: 8 sessions, NR session duration	1) Psychoeducation: sexual response cycle, body image, touching with pleasure, 2) Mindfulness: body scan, mindful eating, mindfulness during a regular activity, stretch and breath, sitting meditation, 3 minutes breathing space, mindfulness of sound, focusing exercise, working with difficulty meditation, stay present during sexual activity handout, mindful standing and walking, mindfulness, self-observation and body sensation, sensate focus, sexual sensation awareness with aids exercise and without different aid, and 3) CBT: snowball worksheet, my four P’s worksheet, list of sexual beliefs, take note of any thoughts and beliefs, taking stock (current, sexual encounters CBT diagram)	Yes	NR	NR	NR
19. Brotto et al. (2022): Canada	NRCT: 8 sessions, NR session duration	1) Psychoeducation: body image, the role of thoughts and belief in sex, introduction to self-touch, pleasurable touch, fantasy erotica and vibrators as sexual stimuli, sexual communication, additional treatment options, 2) Mindfulness: mindful eating, body scan, mindful stretching, walking meditation, mindfulness of thoughts meditation sexual sensation meditation, working with difficulty meditation, sensate focus, and 3) CBT: the role thought and beliefs in sex, positive communication skills, focus on contextual maintaining factors and skill, relapse prevention	Yes	Homework Rating Scale + Ad hoc - Likert scale from 0-10 in two questions: (i) how easy or difficult was it to do the home practice(s)?; (ii) how much of the assignments were you able to complete?	NR	NR
20. Chang et al. (2022): Taiwan	NRCT: MBSR: 6 sessions, 120 min each	1) Mindfulness: sitting meditation, body scan, walking meditation practice exercises with audio-recorded mindfulness practice, and 2) Physiotherapy: bladder and pelvic floor muscle training	NR	NR	± 36	±10-15 daily
21. Donat et al. (2022): USA	NRCT: 8 sessions, NR session duration	1) Psychoeducation: journal exercises, inspirational quotes, and 2) Mindfulness: pre-taped meditation exercises were given with exercises included seated meditation, body scan and comparison meditation, 1 minute mindfulness practice and 3) CBT: practice cards	NR	NR	NR	NR
22. Gorman et al. (2022): USA	NRCT: 8 sessions, between 90-120 min each	1) Psychoeducation: body image, letter to self about the importance of sexuality body image, self-exploration with touch, sexual response cycle, tips for communicating with partners and health-care provider, 2) Mindfulness: mindfulness of breath mindfulness eating, everyday life, focusing exercise, meditation about past, present and future thoughts, mindfulness of sounds, mindful standing and walking, self-observation, staying present during sexual activity, sensory awareness, body awareness, self-observation, sexual sensations awareness meditation, sensate focus, and 3) CBT: snowball exercise about sexual concerns, explore sexual aids, my four P’s worksheet, CBT model worksheet, monitoring sexual beliefs, my thought biases worksheet	Yes	NR	± 56	± 149 weekly
23. Mojtehedi et al. (2022): Iran	RCT: 8 sessions, NR session duration	1) Mindfulness: body scan, mindfulness of breath, meditation of body parts, sounds, thoughts, and conscious choices, accept and allow	NR	NR	±35	NR
24. Nejad et al. (2022): Iran	RCT: 8 sessions, 75 min each	1) Psychoeducation: thinking about the sexual concepts study of the booklet given by the researcher and do one exercise, 2) Mindfulness: everyday life, body scan, mindfulness of breath and walking meditation and sitting meditation, and 3) CBT: monitoring sexual beliefs, record pleasant events of thoughts and feeling, make a list of unpleasant events, write down thoughts and feelings, and bodily sensations related to them	NR	NR	±56	NR
25. Rashedi et al. (2022): Iran	RCT: 4 sessions, 90-120 min each	1) Mindfulness: body scan, seated meditation, sensate focus, and 2) CBT: Identify positive and negative thoughts during sexual activity, writing down undesirable situations, spontaneous thoughts, schemas and behaviors exhibited in each situation	Yes	NR	21	± 37.5 daily
26. Saniei et al. (2022): Iran	RCT: 6 sessions, 90 min each	1) Mindfulness: focusing on the present moment, physical mindfulness, emotional mindfulness, thinking mindfulness, emotion mindfulness, body thought, and emotion mindfulness and mindfulness regimen	NR	NR	±84^c^	NR
27. Jaderek et al. (2023): Poland	NRCT: 4 sessions, 150 min each	1) Mindfulness: body scan, sitting meditation, mindful yoga, interception meditation, sexuality meditation and mindful of breath	NR	Ad hoc - Participants were asked to provide information of the quantity and quality of each meditation presented during meetings, and they documented how often they practiced each assigned exercise every week. Each item was rated on a Likert scale involving a frequency in terms of days: once a week, twice a week, 3 or 4 times a week, more often or never	± 14	±210 weekly
28. Najafabadi et al. (2023): Iran	RCT: 8 sessions, 60 min each	1) Psychoeducation: observing and touch genitals, writing literary prose, stories, and poetry even in the simplest form, 2) Mindfulness: meditation, body scan with and without genitals, mindful walking, sitting and breathing meditation, eating mindfully and guided meditation, and 3) Physiotherapy: Kegel exercise	NR	NR	NR	NR
29. Sears et al. (2023): Canada	NRCT: 8 sessions, 120 min each	1) Psychoeducation: sexual to self about the importance of sexuality, circular response cycle, body image, self-exploration with genitals, touch with pleasure, snowball, my four P’s, 2) Mindfulness: focusing exercise, self-observation, focusing on the present moment, sexual sensation awareness meditation taking stock, sensate focus, and 3) CBT: monitoring sexual beliefs, CTB diamond	Yes	Ad hoc - Questions included a checklist of completed worksheets and exercises and questions relating to amount of mindfulness practice	NR	NR
30. Thomas et al. (2023): Canada	RCT 6 sessions, 120 min each	1) Mindfulness: with audio recordings	NR	NR	36	60 daily

Abbreviations: CBT, cognitive behavioral therapy; ES, education support; M, mindfulness; MBCT, mindfulness-based cognitive therapy; M-gCBT, is a technique that combines meditation with elements of gCBT; MBSR, mindfulness-based stress reduction; NR, not reported; NRCT, nonrandomized clinical trial; PLMEG, pelvic floor exercise group; PVD, provoked vestibulodynia; RCT, randomized clinical trial; WK, weeks.

a Homework duration was estimated according to the data available in each study. For clarity, we standardize the homework duration in several daily sessions.

b The session number and duration refer to what each study researcher recommended, but it is not possible to ensure that the research participants follow these recommendations.

**Table 2:** Characteristics of the participants.

**N°**	**Sample size** ^**a**^	**Age, mean** **(SD), y**	**Sexual dysfunction**
1	36	37 (NR)	Sexual desire/arousal
2	22	49.4 (NR)	Sexual dysfunction in women with gynecologic cancer
3	20	35.8 (NR)	Sexual distress in women with a history of childhood sexual abuse
4	IT: 68	40.8 (11)	Sexual interest/arousal disorder
	DT: 47	42.3 (12.70)	
5	35	44.41 (3.94)	Sexual dysfunction after risk-reducing salpingo-oophorectomy
6	IT: 62	39.0 (13.78)	Provoked vestibulodynia
	DT: 23	40.4 (11.35)	
7	79	49.8 (11.5)	Sexual desire/arousal
8	MBSR: 9	46.4 (15.2)	Sexual dysfunction in women with interstitial cystitis/bladder pain syndrome
	UC: 11	44.4 (13.9)	
9	26	43.9 (12.1)	Sexual interest/arousal disorder
10	SS: 30	40.33 (10.36)	
	MB: 20	39.20 (9.7)	
	WL: 20	37.35 (9.12)	Low sexual desire
11	PLMEG: 32	36 (7.2)	
	M: 23	35.7 (6.8)	
	PLMEG + M: 25	35.5 (5.7)	Sexual dysfunction in women with multiple sclerosis
12	CBT: 63	31.24 (8.99)	Provoked vestibulodynia
	MBCT: 67	33.71 (7.48)	
13	M-gCBT: 14	34.3 (6.2)	Provoked vestibulodynia
	ES: 17	29.1 (6.1)	
14	MBSR: 34	47.7 (5.2)	Sexual dysfunction with menopausal women after risk-reducing salpingo-oophorectomy
	CAU: 31	48.5 (5.4)	
15	MBCT: 35	32.22 (8.48)	Orgasm disorder
	CBT: 32	33.16 (10.65)	
16	20	38.60 (6.58)	Sexual dysfunction in women breast cancer survivors
17	STEP: 78	37.9 (12.2)	Sexual interest/arousal disorder
	MBCT: 70	29.3 (13.2)	
18	5	45 (NR)	Sexual dysfunction in women with cancer
19	30	35.33 (9.67)	Sexual interest/arousal disorder
20	MBSR: 36	50.19 (11.25)	Sexual dysfunction in women with breast cancer
	UC: 25	45.25 (5.90)	
21	20	33 (NR)	Chronic pelvic pain
22	22	53.2 (9.4)	Sexual dysfunction in women cancer survivors
23	MBI-P: 27	56 (3.21)	
	Aroma-MBI: 29	55.34 (3.32)	Sexual dysfunction in postmenopausal women
	RC-P: 29	56.59 (4.35)	
24	I: 22	30.65 (4.76)	Sexual dysfunction in women suffering from infertility
	C: 22	29.45 (5.10)	
25	I: 35	28.5 (6.9)	Sexual interest/arousal disorder
	C: 35	37.0 (7.0)	
26	EG: 32	25.22 (3.50)	Sexual desire in primigravida pregnant women
	CG: 33	25.03 (4.07)	
27	WSD: 53	30.66 (7.43)	Sexual dysfunction
	NSD: 40	31.15 (7.02)	
28	I: 56	28.9 (4.53)	Sexual dysfunction in women with premenstrual syndrome
	C: 56	29.7 (5.3)	
29	30	54.10 (8.27)	Sexual interest/arousal disorder in women following breast cancer treatment
30	M: 31	55.9 (7.1)	Sexual interest/arousal disorder in midlife women
	E: 30	55.7 (7.5)	

Abbreviations: Aroma-MBI, aromatherapy–mindfulness-based intervention; Aroma-RC, aromatherapy–routine care; C, control; CAU, care as usual; CBT, cognitive behavioral therapy; CG, control group; DT, delayed treatment; E, educational; EG, experimental group; ES, education support; HC, homework compliance; I, intervention; IT, immediate treatment; M, mindfulness; MBCT, mindfulness-based cognitive therapy; M-gCBT, meditation combined with elements of gCBT; MB, mindfulness-based group; MBI-P, mindfulness-based intervention–placebo; MBSR, mindfulness-based stress reduction; MBCT, mindfulness-based cognitive therapy; MBT, mindfulness-based therapy; NR, not reported; NSD, no sexual dysfunction; PLMEG, pelvic floor exercise group; RC-P, routine care-placebo; SS, scheduled sex and motivations group; STEP, supportive sex education and therapy group; UC, usual care; WL, waiting-list; WSD, with sexual dysfunction.

a Sample size was reported according to the study description. For example, in cases where only the total mean age was reported, we recovered only the sample total n and mean age. In cases where the group reported the mean age, this information was retained.

### Characteristics of the included studies and their participants

Almost half (n *=* 12) of the papers included in our review were conducted in Canada, followed by the United States (n *=* 7) and Iran (n *=* 6). Researchers from Poland, Belgium, Finland, Taiwan, and the Netherlands conducted 1 study each. There were equal numbers of RCT (n *=* 15) and NRCT (n *=* 15) studies. The included articles were published between 2008 and 2023; most (n *=* 16) were published in the last 5 years.

Regarding the types of sexual dysfunction investigated, studies of sexual interest/arousal disorder were the most frequent (n *=* 10), followed by studies related to female survivors of cancer (n *=* 6); women with provoked vestibulodynia (n *=* 3), orgasm disorder (n *=* 1), or chronic pelvic pain (n *=* 1); specific populations such as women who were postmenopausal (n *=* 1), had sexual distress and a history of childhood abuse (n *=* 1), had undergone risk-reducing salpingo-oophorectomy (n *=* 2), had interstitial cystitis/bladder pain syndrome (n *=* 1), had multiple sclerosis (n *=* 1), or suffered from infertility (n *=* 1), premenstrual syndrome (n *=* 1), or nonspecified sexual dysfunction.

Treatment duration ranged from 1 to 16 sessions, but almost 50% of the studies (n *=* 14) were conducted using 8 sessions of MBCT. The same variety can be seen in the minutes used in each intervention session, since the shortest session was 60 minutes and the longest was 240 minutes. In some studies (n *=* 7) the session duration for each intervention was not reported.

The sample sizes of the included studies ranged from 5 to 148 participants, with a total of 1747 women participating in the 30 studies. It was not possible to calculate the overall mean age of all studies as some did not report the mean, and many did not report the SD. Despite this lack of reporting, we found that between the studies that reported mean (SD) age, the lowest reported values were 25.1 (3.7) and the highest were 55.83 (10.3).

### Homework prescribed

Mindfulness exercises, such as body scan, sitting mindfulness, eating mindfulness, breathing mindfulness, stretch and breath, self-observation, and sensate focus (a couples-based mindfulness exercise) were most frequent homework prescribed (n *=* 28). The second most common homework prescribed was psychoeducation (n *=* 17), such as reading, performing body image exercises, learning about the sexual response cycle, and touching with pleasure. Less frequently prescribed homework was individual or couple CBT (n *=* 15), including worksheets, communication, behavior exercises, monitoring sexual beliefs, progressive muscle relaxation, and taking stock of sexual concerns. Physiotherapy exercises (n = 2), such as Kegel exercises and pelvic movement, were also prescribed in some studies.

Partners were asked to participate in homework in only 9 of the analyzed studies. Across these studies, psychoeducation, mindfulness, and CBT homework were prescribed equivalently in 7 studies. Only one study prescribed psychoeducation, and another mindfulness plus CBT as homework.

### Instruments used to assess homework adherence

Most studies (n = 21) did not report how homework adherence was assessed, even though the publication noted that homework was assessed. Some studies used only ad hoc instruments (n *=* 6) to measure homework adherence. In this evaluation, Likert scales ranging from 0 to 2 were used to ask whether or not the participants had completed the homework and whether they had noticed a difference in improving their symptoms after completing these homework activities (n *=* 3). In 1 study (n *=* 1), a Likert Scale from 1 to 5 was used to assess homework adherence. In another study (n = 1) participants were also asked to complete a checklist of worksheets, exercises, and questions relating to the amount of mindfulness practice. In another study (n *=* 1) participants were asked to provide information on the quantity and quality of each meditation presented during the sessions and to document how often the participants practiced each assigned exercise each week. This Likert scale was based on number of days per week: once a week, twice a week, 3 or 4 times a week, more often, or never.

Two studies used both instruments with good psychometric validity and ad hoc items. One of them used a Likert Scale from 0 (not at all) to 4 (high degree) to verify to what extent the participants were able to complete the homework tasks and to what extent they were able to practice the skills and exercises they had learned in the group. In addition to the ad hoc items, the authors also used the MBCT Adherence Scale (MBCT-AS), which is based on a 17-item scale that was aimed to measure therapist adherence to the treatment protocol for MBCT to address recurrence of major depressive disorder. This scale was modified by Brotto et al,^43^^,^^44^ and just 7 items were used. These studies also used two ad hoc questions to assess homework: (1) “Overall, to what degree were you able to complete your homework assignments?” and (2) “Overall, since the last group session, to what degree have you been able to practice the skills and exercises you learned in group?”

A different study^17^ used 2 ad hoc questions in a Likert Scale from 0 to 10: (1) “How easy or difficult was it to do the home practice(s)?” and (2) “How many of the assignments were you able to complete?” In addition, the authors used the Homework Rating Scale, which is a 12-item self-report measure created to measure the therapist, client, and task characteristics between client compliance with homework assignments using a 5-point scale. This study^17^ was the only study in which a psychometric instrument (the MBCT Adherence Scale) was used exclusively to assess homework adherence.

## Discussion

In this scoping review we investigated the most commonly used instruments for assessing homework adherence in interventions evaluating MBCT for FSD and the types of homework used in these interventions. Our main findings suggest that most publications do not provide information on how they assessed homework adherence. Therefore, there is no gold standard instrument for measuring homework adherence during MBCT treatment for FSD.

Reports of 9 studies that described how homework was assessed used only ad hoc measures. However, it is noteworthy that the use of nonvalidated instruments is something that should be avoided because such instruments may lead to mistaken conclusions and difficulty in comparing results with other studies.^33–36^ Furthermore, the creation of a new measurement instrument, even with good psychometric evidence, must be based on a sound theoretical foundation that justifies its need. For this reason, the literature review and the confirmation that there are no similar existing instruments are the first steps recommended in some best practice guidelines for instrument development.^33^^,^^36^

Despite the lack of standardization in how homework adherence is assessed in trials evaluating MBCT treatment for FSD, there are instruments available in the field of CBT to measure homework in a variety of conditions, such as the Homework Rating Scale–II Client and Therapist versions for depression,^45^^,^^46^ and the Patient Exposure and Response Prevention Adherence Scale—PEAS^47^ for obsessive-compulsive disorder. However, previous meta-analyses in the field of CBT have shown that there are few psychometric instruments available to measure homework on the basis of the client point of view and that it is common to use instruments that assess the therapist’s skills and competence in engaging the participant in homework.^48^ Furthermore, many of the available instruments, such as the PEAS,^47^ specifically assess certain techniques and/or contexts associated with specific psychopathology (eg, anxiety, obsessive compulsive disorder, attention deficit hyperactivity disorder). Therefore, even in the field of CBT there is a lack of instruments available to measure homework in cases of FSD.^48^

Among the questions included in the ad hoc instruments, most of them asked the participants about whether homework was completed, as well as if the participants noticed an improvement in symptoms as a result of doing the homework; to what degree they were able to complete the homework; to what degree they were able to practice the skills and exercises learned in the group; how easy or difficult it was to do the practice at home; and how clear the instructions were for doing the homework. The findings are similar to those pointed out by previous authors that showed that few studies used psychometric instruments in psychotherapy to access homework compliance, making it possible only to verify the quantity (or extent) of homework compliance rather than the quality of learning associated with home practices or th therapist’s ability in engaging participants for homework practice.^48–50^

The most frequently prescribed homework exercises were mindfulness exercises, followed by psychoeducation, and then CBT skills (that were included as part of an MBCT program). Thus, our findings are aligned with those reported in the literature, which point to these techniques as the most effective for treating FSD.^7^^,^^43^^,^^51^ In the field of human sexuality, interventions based on mindfulness have proven to be effective in the treatment of both female and male sexual dysfunction, mainly because these techniques help participants to cultivate nonjudgment and increased observation of bodily sensations and emotions, as well as self-compassion.^15^^,^^23^^,^^43^^,^^52^ Psychoeducation has also been proven to be effective for improving sexual function and response. The work performed by Masters and Johnson^53^ on sensate focus showed how knowledge of sexuality could minimize sexual suffering. Understanding the sexual response cycle, performing body image exercises, and addressing questions about the evaluation of factors that contribute to sexual experience, as well as sensate focus (which is now considered to be a mindfulness exercise) help to reduce anxiety and increase understanding of the physiology of the sexual act, favoring a healthier experience of sexual relations and bodily functioning.^31^^,^^54^^,^^55^ Last, CBT techniques, such as self-monitoring, evaluating thoughts, working with automatic thoughts and beliefs, and cognitive restructuring, have also proved to be effective during FSD treatment.^7^^,^^51^ Homework exercises that involve a partner are also often part of couples therapy programs aimed at resolving marital conflicts and for managing FSD.^56–58^

The current review is novel because it integrates reports of homework adherence measures with studies on FSD that have evaluated mindfulness, and overall, we found that there was no gold standard measure to assess homework adherence. Despite its strengths, this review has some limitations. First, the aim of our study was the investigation of MBCT interventions that prescribed homework. Despite this focus, some of the included studies used both MBCT and CBT as interventions and homework. Consequently, our results should not be generalized to other interventions. Second, most of the studies took place in Western countries, and the findings cannot be generalized to all cultures. Finally, most of the included studies did not report which instruments were used to analyze homework adherence, revealing a significant gap in this field of study. This gap is even more curious, given that several previous studies have demonstrated the importance of homework activities in acquiring new behavioral habits and consequently reducing the sexual symptoms.^23^^,^^52^^,^^59^ Despite that, our scoping review concludes that among the studies that reported how homework adherence was assessed, most used ad hoc measures.

Overall, although there are some psychometric instruments available to evaluate homework in the CBT field, when it comes to evaluating homework adherence with MBCT for FSD, most investigators are relying on ad hoc instruments. It is important that in future research, investigators seek to adapt the instruments available in the field of CBT and/ to measure homework adherence in the field of FSD. Another possibility is the development of psychometrically validated instruments to specifically assess homework adherence in the FSD research field. In line with the recommendations of Kazantzis and Miller,^50^ we also recommend that new homework adherence instruments should consider both quantitative (ie, amount of adherence) and qualitative (ie, quality of the homework and therapist skills in engaging clients in homework practice) aspects during homework assessment.

Finally, clinicians involved in treatment outcome research should not only prescribe homework to trial participants, but also monitor the progress of these activities. Discussing and evaluating the assigned activities with trial participants allows for the emotional, cognitive, and behavioral skills developed in therapy to be practiced between sessions. This practice helps integrate these skills into the client’s emotional and behavioral repertoire, thereby facilitating the necessary changes for well-being and sexual health. Therefore, although our review concludes that there is a lack of validated measures of homework adherence, we identified the existence of three instruments with psychometric evidence that can be used by clinicians to monitor homework adherence.^25^

## Supplementary Material

Supplementary_Material_qdae108
