# Supplementary material for: Homework adherence in mindfulness-based cognitive interventions for female sexual dysfunction: a scoping review
Source: J Sex Med. 2024 Sep 12;21(11):1064–75. doi: 10.1093/jsxmed/qdae108 (PMC11534372; doi:10.1093/jsxmed/qdae108)
Supplement: Supplementary_Material_qdae108 [file supplementary_material_qdae108.docx]

**Supplementary Material**

Articles included and analyzed in this review

1- Brotto LA., Basson L, Luria M. A mindfulness-based group psychoeducational intervention targeting sexual arousal disorder in women. J Sex Med. 2008. 5:1646–1659. doi: 10.1111/j.1743-6109.2008.00850.x

2- Brotto LA, Heiman JR, Goff B, Greer B, Lentz GM, Swisher E, et al. A psychoeducational intervention for sexual dysfunction in women with gynecologic cancer. Arch Sex Behav. 2008 Apr;37(2):317-29. doi: 10.1007/s10508-007-9196-x​

3- Brotto LA, Seal BN, Rellini A. Pilot study of a brief cognitive behavioral versus mindfulness-based intervention for women with sexual distress and a history of childhood sexual abuse. J Sex Marital Ther. 2012;38(1):1-27. doi:10.1080/0092623X.2011.569636​

4- Brotto LA, Basson R. Group mindfulness-based therapy significantly improves sexual desire in women. Behav Res Ther. 2014;57:43-54. doi:10.1016/j.brat.2014.04.001

5- Bober SL, Recklitis CJ, Bakan J, Garber JE, Patenaude AF. Addressing sexual dysfunction after risk-reducing salpingo-oophorectomy: Effects of a brief, psychosexual intervention. J Sex Med. 2015;12:189-197.

6- Brotto LA, Basson R, Smith KB, Driscoll M, Sadownik L. Mindfulness-based group therapy for women with provoked vestibulodynia. Mindfulness. 2015;6(3):417–432. doi: 10.1007/s12671-013-0273-z

7- Brotto LA, Chivers ML, Millman RD, Albert A. Mindfulness-based sex therapy improves genital-subjective arousal concordance in women with sexual desire/arousal difficulties. Arch Sex Behav. 2016;45(8):1907–1921. doi: 10.1007/s10508-015-0689-8

8- Kanter G, Komesu YM, Qaedan F, Jeppson PC, Dunivan GC, Cichowski SB, Rogers RG. Mindfulness-based stress reduction as a novel treatment for interstitial cystitis/bladder pain syndrome: a randomized controlled trial. Int Urogynecol J. 2016;27(11):1705–1711. Doi: 10.1007/s00192-016-3022-8

9- Paterson LQP, Handy AB, Brotto LA. A pilot study of eight-session mindfulness-based cognitive therapy adapted for women’s sexual interest/arousal disorder. J Sex Res. 2017;54(7):850–861. doi: 10.1080/00224499.2016.1208800

10- Gunst A, Ventus D, Arver S, Dhejne C, Görts-Öberg K, Zamore-Söderström E, Jern P. A randomized, waiting-list-controlled study shows that brief, mindfulness-based psychological interventions are effective for treatment of women’s low sexual desire. J Sex Res. 2018;55(3):353–365. doi: 10.1080/00224499.2018.1539463

11- Mosalanejad F, Afrasiabifar A, Zoladl M. Investigating the combined effect of pelvic floor muscle exercise and mindfulness on sexual function in women with multiple sclerosis: a randomized controlled trial. Clin Rehabil. 2018;32(10):1340–1347. doi: 10.1177/0269215518775787

12- Brotto LA, Bergeron S, Zdaniuk B, et al. A comparison of mindfulness-based cognitive therapy vs cognitive behavioral therapy for the treatment of provoked vestibulodynia in a hospital clinic setting. J Sex Med. 2019;16(7):909–923. doi: 10.1016/j.jsxm.2019.04.002.

13- Guillet AD, Cirino NH, Hart KD, Leclair CM. Mindfulness-based group cognitive behavior therapy for provoked localized vulvodynia: a randomized controlled trial. J Lower Genital Tract Dis. 2019;23(2):119–125. doi: 10.1097/LGT.0000000000000456

14- van Driel CMG, de Bock GH, Schroevers MJ, Mourits MJ. Mindfulness-based stress reduction for menopausal symptoms after risk-reducing salpingo-oophorectomy (PURSUE study): a randomised controlled trial. BJOG. 2019;126(10):1248–1256. doi: 10.1111/1471-0528.15471

15- Adam F, Sutter P, Day J. et al. A Randomized Study Comparing Video-Based Mindfulness-Based Cognitive Therapy With Video-Based Traditional Cognitive Behavioral Treatment in a Sample of Women Struggling to Achieve Orgasm. J Sex Med. 2020; 17:312e324. doi: 10.106/j.jsxm.2019.10.022

16- Bober SL, Fine E, Recklitis CJ. Sexual health and rehabilitation after ovarian suppression treatment (SHARE-OS): a clinical intervention for young breast cancer survivors. J Cancer Surviv. 2020;14(1):26–30. doi: 10.1007/s11764-019-00800-x.

17- Brotto LA, Zdaniuk B, Chivers ML, Jabs F, Grabovac A, Lalumière ML, Weinberg J, Schonert-Reichl KA, Basson R. A randomized trial comparing group mindfulness-based cognitive therapy with group supportive sex education and therapy for the treatment of female sexual interest/arousal disorder. J Consult Clin Psychol. 2021;89(7):626–639. doi: 10.1037/ccp0000661.

18- Gorman JR, Drizin JH, Al-Ghadban FA, Rendle KA. Adaptation and feasibility of a multimodal mindfulness-based intervention to promote sexual health in cancer survivorship. Transl Behav Med. 2021;11(6):1885–1895. doi: 10.1093/tbm/ibab083

19- Brotto LA, Stephenson KR, Zippan N. Feasibility of an online mindfulness-based intervention for women with sexual interest/arousal disorder. Mindfulness. 2022;13(3):647–659. doi: 10.1007/s12671-021-01820-4

20- Chang YC, Lin GM, Yeh TL, Chang YM, Yang CH, Lo C, Yeh CY, Hu WY. Impact of mindfulness-based stress reduction on female sexual function and mental health in patients with breast cancer. Support Care Cancer. 2022;30(9):4315–4325. doi: 10.1007/s00520-021-06540-y.

21- Clark Donat LE, [Outros autores]. The effects of a brief mindfulness-based intervention on pain perceptions in patients with chronic pelvic pain: a case series. Case Rep Womens Health. 2022;33. doi: 10.1016/j.crwh.2021.e00380.

22- Gorman JR, Drizin JH, Smith E, et al. Feasibility of Mindful After Cancer: pilot study of a virtual mindfulness-based intervention for sexual health in cancer survivorship. J Sex Med. 2022;19(6):1131–1146. doi: 10.1016/j.jsxm.2022.03.618

23- Mojtehedi M, Salehi-Pourmehr H, Ostadrahimi A, Asnaashari S, Esmaeilpour K, Farshbaf-Khalili A. Effect of aromatherapy with essential oil of Lavandula angustifolia Mill. and Citrus bergamia and mindfulness-based intervention on sexual function, anxiety, and depression in postmenopausal women: a randomized controlled trial with factorial design. Iran J Nurs Midwifery Res. 2022;27(5):392–405. doi: 10.4103/ijnmr.ijnmr_129_21.

24- Hosseini NS, Bokaie M, Yassini ASM. Effectiveness of sexual health counseling based on mindfulness approach on sexual satisfaction in women suffering from infertility: an RCT. Int J Reprod BioMed. 2023;21(2):147–158. doi: 10.18502/ijrm.v21i2.12805

25- Rashedi S, Maasoumi R, Vosoughi N, Haghani S. The effect of mindfulness-based cognitive-behavioral sex therapy on improving sexual desire disorder, sexual distress, sexual self-disclosure, and sexual function in women: a randomized controlled clinical trial. J Sex Marital Ther. 2022;48(5):475–488. doi: 10.1080/0092623X.2021.2008075

26- Saniei S, Fahami F, Samouei R, Ghasemi Tehrani H. Investigation of the effect of mindfulness on sexual desire and sexual satisfaction in primigravida pregnant women. J Educ Health Promot. 2022;11:61. doi: 10.4103/jehp.jehp_176_21

27- Jąderek I, Obarska K, Lew-Starowicz M. Assessment of the effect of mindfulness monotherapy on sexual dysfunction symptoms and sex-related quality of life in women. Sex Med. 2023;11(1):1–17. doi: 10.1093/sexmed/qfad022

28- Najafabadi SH, Vakilian K, Ghaemmaghami M, Zamanian M, Beigi M. Investigating the effect of mindfulness counselling on sexual functioning of women with premenstrual syndrome. Sex Reprod Healthc. 2023;37:100886. doi: 10.1016/j.srhc.2023.100886.

29- Sears C, Millman R, Brotto LA, Walker LM. Feasibility and acceptability of a group-based mindfulness intervention for sexual interest/arousal disorder following breast cancer treatment. J Sex Marital Ther. 2023;49(5):533–549. doi: 10.1080/0092623X.2022.2154296

30- Thomas HN, Brotto LA, Cameron FDA, Yabes J, Thurston RC. A virtual, group-based mindfulness intervention for midlife and older women with low libido lowers sexual distress in a randomized controlled pilot study. J Sex Med. 2023;20(7):1060–1068. doi: 10.1093/jsxmed/qdad081
